# Identification of Misplaced Endometrial Glands and Stroma in the Myometrium of Foetal Uteri Evocative of Developmental Adenomyosis

**DOI:** 10.3390/ijms27083595

**Published:** 2026-04-17

**Authors:** Jean Gogusev, Yves Lepelletier, Nicolas Lopez, Patrick Barbet, Pierre Validire

**Affiliations:** 1 Institut Cochin, INSERM UMR 1016, CNRS 8104, Université Paris Cité, 22 Rue Méchain, 75014 Paris, France; 2 W-MedPhys, 128 Rue la Boétie, 75008 Paris, France; yves.lepelletier@u-paris.fr (Y.L.); nicolas.lopez2@ipht.fr (N.L.); 3 Institut Imagine, Université Paris Cité, 24 Boulevard Montparnasse, 75015 Paris, France; 4 INSERM UMR 1163, Laboratory of Cellular and Molecular Basis of Normal Hematopoiesis and Hematological Disorders: Therapeutical Implications, 24 Boulevard Montparnasse, 75015 Paris, France; 5 ENOES/ENESIA, 62 Rue de Miromesnil, 75008 Paris, France; 6 Unité Mixte de Recherche “Institut de Physique Théorique (IPhT)” CEA-CNRS, UMR 3681, Route de l’Orme des Merisiers, 91191 St Aubin-Gif-sur-Yvette, France; 7 Service d’Anatomie Pathologique, Hôpital Universitaire Necker-Enfants Maladies, 149 Rue de Sèvres, CEDEX 15, 75743 Paris, France; barbet.jp@gmail.com; 8 Service d’Anatomie Pathologique, Institut Mutualiste Montsouris, 42 Bd Jourdan, 75014 Paris, France; pierre.validire@orange.fr

**Keywords:** adenomyosis, foetal uteri, monozygotic twins’ (MZ), developmental adenomyosis, myometrium, stromal layer, smoot muscle, hyperplasia/hypertrophy

## Abstract

Adenomyosis is a benign gynaecological disorder in which endometrial glands and stroma enter the uterine myometrium with varying degrees of spreading. To analyse the presence of developmentally displaced endometrial glands and stroma in the foetal myometrium, a retrospective cohort of 420 foetal uteri, including one monozygotic twin pair, was histopathologically evaluated. The gestational age ranged between 18 and 37 weeks; the clinical characteristics included various foetal malformations with a predominantly normal karyotype, except in one case with trisomy 18. Ectopic endometrial tissue enclosed in the myometrium was discovered in twelve individual foetuses from the cohort (12/420). The investigation of the histogenetic attributes of the misplaced endometrial tissue in both monozygotic twins’ (MZ) foetal uteri revealed isolated glands and thin channels containing cords of endometrial-type glands penetrating the myometrium. Through immunohistochemistry, low levels of oestrogen receptors (ERs) were detected, whereas a moderate level of progesterone receptor (PR) expression was observed in the ectopic glandular and stromal cell nuclei in all cases. Additionally, the surrounding periglandular component consistently expressed the vimentin and CD10 stromal cell markers, while the myometrial smooth muscle cells revealed the strong expression of both alpha-Smooth Muscle Actin (α-SMA) and desmin marker proteins.

## 1. Introduction

Uterine adenomyosis, also known as endometriosis interna, is a complex disease characterised by the presence of endometrial glands and stroma developed within the myometrium [1,2,3,4]. Two main pathogenetic theories have been proposed to explain the origin of the disease. The first concept proposes invagination of the endometrial basalis into the myometrium, causing the activation of the tissue injury and repair mechanism [3,5,6]. The alternative theory suggests de novo development of adenomyosis after metaplastic changes of the pluripotent Müllerian mesonephric remnants present in the myometrium or differentiation of progenitor stem cells [2,7,8].

The theory of Müllerianosis predicts that embryonic tissue is misplaced during organogenesis, resulting in the delayed formation of four benign Müllerian diseases, including developmental adenomyosis, endometriosis, endosalpingiosis, and endocervicosis [7]. Additional studies have likewise suggested that endometriosis, a closely related disorder to adenomyosis, arises from primitive endometrial tissue misplaced along the migratory pathway during embryogenesis [9,10,11]. These studies assessed that the incidence of the dislocated embryonic structures found through the foetal pelvic cavity is similar to that of endometriosis occurring in the adult female population [9]. Similarly, displaced endometrial glands and embryonic duct remnants were identified along the female foetal reproductive tract, potentially related to different benign and neoplastic pathological conditions [12].

On the other hand, a causal relationship between exposure to the estrogenic endocrine disruptor Diethylstilbestrol (DES) and the development of adenomyosis was confirmed by studying experimental models of hybrid female mice [13,14]. Moreover, the endocrine disruptor Bisphenol A elicits more severe pathologies in female mice, including adenomyosis, leiomyomas, atypical hyperplasia, and stromal polyps [15].

Clinically, adenomyosis in adults is considered a heterogeneous disease, with its diagnosis being difficult as it requires a concerted evaluation of clinical parameters, imaging procedures, and histopathological characteristics [11,16]. In this regard, a significant correlation between response to treatment and histological variants of adenomyosis has been demonstrated by several studies, highlighting the importance of having a consonant standardised classification system for directing treatment [17,18,19,20].

However, at present, there is a lack of uniformly validated image-based classification systems in accordance with the histopathologic definitions that can guide accurate clinical decisions [21,22]. Applying magnetic resonance imaging, four subtypes of adenomyosis can be delineated: subtype I—intrinsic (inner myometrium), subtype II—extrinsic (outer myometrium), subtype III—intramural (surrounded by normal outer myometrium), and subtype IV—indeterminate (not fitting in any of the other types) [23].

The histological standardisation of adenomyosis in adults is primarily based on the depth of penetration and degree of spread of the ectopic tissue along the myometrium [24]. In agreement with these parameters, the adenomyotic lesions are classified as grade I invading the subendometrial basalis, grade II up to the mid-myometrium, and grade III beyond the mid-myometrium with a slight, moderate, or marked degree of involvement [24].

With regard to the incidence of adenomyosis, largely variable ratios ranging between 5 and 70% are reported in the literature [25]. These variations are mostly dependent on the procedure and how thoroughly the uterus is sampled during the pathological analysis, i.e., the more sections taken from a given specimen, the higher the rate of finding adenomyotic islands in the myometrium [24]. The minimal distance necessary for histological characterisation ranges from 2 mm to more than 4 mm from the basal endometrium, or from one to two microscopic low-power fields [25]. In fact, when a small number of routine sections of adult uteri were analysed, 31% of hysterectomy specimens contained adenomyosis, but with more than six sections, the rate increased to 61% [24]. Also, using more stringent diagnostic criteria, e.g., penetration deeper than 25% of the myometrial thickness, yielded lower adenomyosis rates than if more superficially located glands were identified. Even in the latter cases, the incidence rates range from 19% [26] in hysterectomy specimens to 57% in uteri examined at autopsy [1,6,27].

In the present study, we evaluated the incidence, anatomical location, and histopathological characteristics of displaced endometrial-type glands and stromal tissue in foetal uterine myometrium from a retrospective cohort composed of 420 cases. Remarkably, the extended assessment revealed the presence of ectopic tissue in the myometrium of 12 foetal uteri from the 420 examined (2.8% of cases), the histopathological characteristics being reminiscent of the adult women’s adenomyotic disorder. In parallel, an analysis of the histogenetic attributes of a monozygotic twin pair showed an analogous distribution of displaced endometrial-type glandular and stromal tissue in the myometrium of both foetal uteri, suggesting a genetically guided developmental process.

## 2. Results

**Figure 1:**
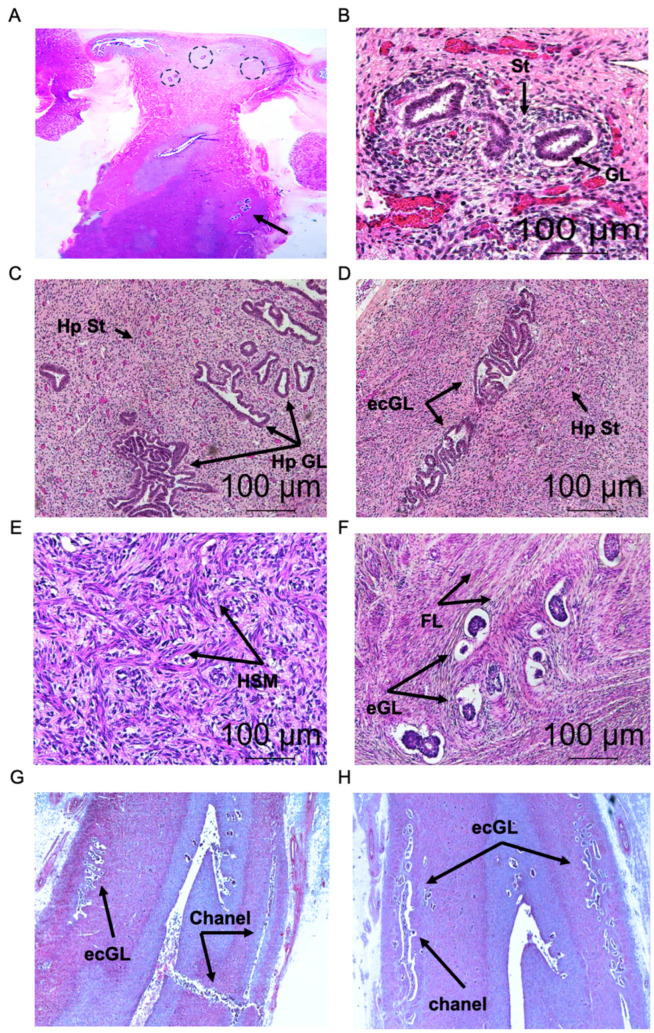
Histological appearance of ectopic endometrial glands and stroma distributed in foetal myometrium. Case no. 8: aspect of a coronal section of the 27-week-old foetal uterus showing displaced ectopic glands and stroma (H&E) (arrows and dotted circles, respectively) (**A**) (magnification ×2.5). Case no. 3: displaced glands in the myometrium surrounded by stromal cells and microvessels (H&E) (arrows show glands (GL) and stroma (St)) (**B**) (magnification ×63). Case no. 2: aspect of hyperplastic ectopic glands in the myometrium surrounded by dense stromal layer containing numerous microvessels (H&E) (arrows show hyperplasic glands (Hp GL) and hyperplasic stroma (Hp St)) (**C**) (magnification ×63). Case no. 7: area of cluster of ectopic convoluted glands surrounded by hyperplastic stromal layer (H&E) (arrows show ectopic convoluted glands (ecGL) and hyperplasic stroma (Hp St)) (**D**) (magnification ×63). Case no. 11: smooth muscle cell hyperplasia at distance from the ectopic glands in the myometrium, showing hypertrophied and disarrayed smooth muscle fibres (H&E) (arrows show hyperplasic smooth muscle area (HSM)) (**E**) (magnification ×63). Case no. 7: strongly developed fibrous layer surrounding clusters of ectopic glands located under the serous uterine surface (H&E) (arrows showing ectopic glands (eGL) and the fibrous layers (FL)) (**F**) (magnification ×63). Case no. 9 and no. 10: sagittal sections of the two individual MZ twins’ foetal uteri exhibiting lengthy slits containing cords of ectopic glands (H&E) (arrows showing ectopic convoluted glands (ecGL) and channels) (**G**,**H**) (magnification ×10).

The presence of misplaced endometrial-type glands and stroma in the foetal uterine myometrium was histologically discovered in 12 cases from the cohort of 420 samples. Approximately 15–20% of the noninformative samples (408 cases) showed mostly the presence of isolated distorted ectopic glands randomly distributed along the inner and outer myometrium, depicted by circles (Figure 1A), without apparent histological changes. The incidence in all informative cases was assessed by quantification of the number of ectopic foci throughout the inner, middle, and outer uterine myometrium. Importantly, no apparent correlation was found between the presence of ectopic glandular foci in relation to the clinical anomalies described in Table 1.

On histological analysis of coronal tissue sections of the whole uteri, ectopic glands with variable morphology and multivariate number were found in the myometrium at different depths of penetration, mainly distributed in the fundus and corpus uteri in all informative cases. The majority of the isolated or clustered misplaced glands in the myometria were often surrounded by variable whitish aureoles corresponding to a dense stromal component, as observed in foetus case no. 8 (Figure 1A; dotted circles). The arrangement of the ectopic foci distributed through the myometrium was histologically examined at a minimal distance of 4 mm from the border of the endometrial junctional zone (JZ), i.e., the compacted subendometrial layer of smooth muscle cells containing little extracellular matrix [1]. Notably, the number of ectopic islands, their structural aspect, the anatomical location, and the depth of penetration in the myometrium were often variable in each informative case (Figure 1B).

In general, the histological analysis of the misplaced endometrial tissue from the various uterine locations predominantly showed glandular forms lined by a single layer of cuboidal epithelial cells without mucinous content in the cytoplasm surrounded by a loose stromal component (Figure 1B). Often, the misplaced glandular structures exhibited disordered cellular polarity, nuclear enlargements, irregular cell shape, and visible nucleolus in all informative cases (Figure 1B). Significantly, clusters of endometrial-type glands with variable number and variable morphology showing mild to moderate hyperplasia were detected in the middle and outer myometrium in all informative cases (Figure 1C). Importantly, the glandular-to-stromal ratio in the ectopic islets appeared variable in all cases without the presence of mitotic figures. In three informative cases (foetal uteri cases no. 5, 6, and 12) (Table 1), the ectopic glandular forms appeared as long-sized encapsulated structures, some being isolated, convoluted and lengthy, distributed at different depths of penetration in the middle and outer myometrium (Figure 1D). Moreover, intense stromal cell hyperplasia displaying an increased number of spindle-shaped cells and microvessels around the displaced glands was revealed through the myometrium in four informative foetal uteri (cases no. 3, 5, 6, and 11) (Table 1). An area of ectopic convoluted glands surrounded by a compact hyperplastic stromal layer in case no. 11 is presented in Figure 1D.

Concerning the smooth muscle component, heterogeneous areas with increased density of smooth muscle fibres adjacent to the misplaced glandular foci, or at a distance from them, were histologically revealed through the middle and outer myometrium in four informative foetal uteri (cases no. 3, 5, 6, and 7) (Table 1 and Figure 1E). An isolated area of smooth muscle fibres in the myometrium at a distance from the ectopic glands, which appeared strongly hypertrophied, disarrayed, and intermingled with fibroblastic-type cells, is presented in Figure 1E.

Less frequently, a thick fibrous component surrounding the displaced glandular structures distributed as isolated or grouped clusters was located mostly along the middle myometrium in two cases (Figure 1F). Histologically, the observed periglandular fibrous tissue exhibits an increased cellularity and is mainly composed of mixed cell populations, including fibroblasts, myofibroblasts, and epithelioid cells (foetal uteri cases no. 7 and 8) (Figure 1F).

The microscopic analysis of the two monozygotic twins’ uteri revealed the presence of isolated or grouped small-sized ectopic glands located in the myometrium of the fundus and the corpus uteri. In particular, numerous lengthy and twisting thin channels filled with glandular structures, which were morphologically small in size, disposed in cords were observed in both uteri (Figure 1G,H).

**Figure 2:**
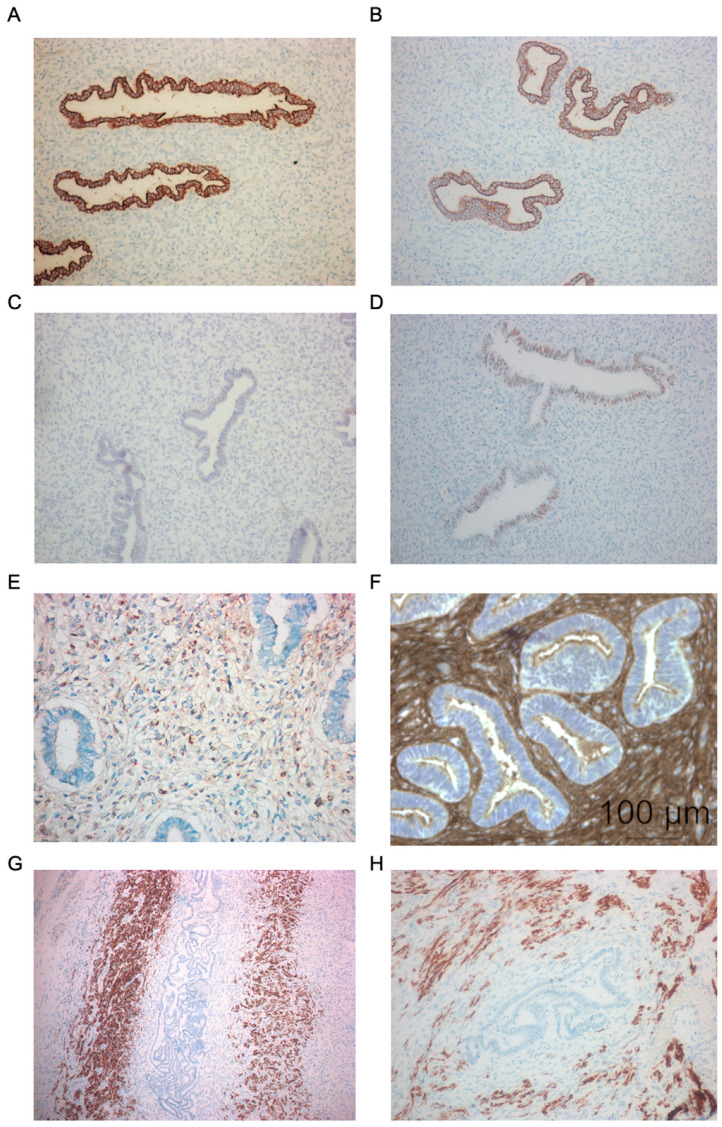
Immunohistochemical (IHC) staining of ectopic glands, stromal layer, and the smooth muscle cells in the foetal myometrium. Immunolabelling with anti-cytokeratin antibody of ectopic hyperplasic glands located under the serosal surface of the myometrium (gland lining epithelial cells coloured in brown). Case 1 (**A**) (magnification ×25), ectopic glands lining epithelial cells stained with anti-EMA antibody (coloured in brown). Case 6 (**B**) (magnification ×25), immunostaining of the nuclear ER-α receptor expression (brown precipitates). Case 11 (**C**) (magnification ×25), staining with anti-PR (A and B forms) antibody (brown precipitates). Case 12 (**D**) (magnification ×25), periglandular hyperplastic stromal layer immunostained with anti-CD10 antibody (brown precipitates). Case 8 (**E**) (magnification ×45), cluster of ectopic glands stained with anti-vimentin antibody (brown precipitates). Case 12 (**F**) (magnification ×45), tissue section of the corpus uteri with area showing immunostaining with anti-α-SMA antibody of hyperplastic smooth muscle fibres (brown precipitates) surrounding an ectopic coiled gland from case 12 (in blue). (**G**) magnification ×10, immunostaining with anti-desmin antibody of the hyperplastic smooth muscle layer (brown precipitates) surrounding a convoluted ectopic gland. Case 7 (**H**) (magnification ×25).

The immunohistochemical analysis of the misplaced, distorted, elongated, and dilated glandular structures showed intense staining for both epithelial markers, cytokeratin (CK) and epithelial membrane antigens (EMA), in all informative cases (Figure 2A,B). The nuclear oestrogen receptor (ER-α) was weakly expressed in both the glandular structures and the surrounding stroma in all informative cases (Figure 2C). In contrast, the nuclear progesterone steroid receptor isoforms PR-A and PR-B were moderately expressed in both the nuclei of the ectopic glandular epithelial cells as well as in the surrounding stromal component (Figure 2D, Table 2).

Significant immunoreactivity for both CD10 and vimentin cell markers was observed in the surrounding stromal layer of the displaced glands in all informative cases (Table 2). Representative immunolabelling with anti-CD10 antibody of a section from the foetal uterus case no. 3 (Figure 2E) and immunostaining with anti-vimentin antibody of a section from the foetal uterus case no. 7 are shown in Figure 2E and Figure 2F, respectively.

The histologically normal as well as the hyperplastic myometrial smooth muscle fibres surrounding the ectopic glands and stroma exhibited strong cytoplasmic immunolabelling for both actin (α-SMA) and desmin proteins, appearing as paranuclear brown-coloured granules in all informative cases (Figure 2G,H) (Table 2).

The immunophenotypic profile of the glandular structures in the monozygotic twins’ (MZ) uteri (cases no. 9 and 10) was generally similar to that observed in the series of informative cases. Indeed, strong immunolabelling for cytokeratin (CK) and epithelial membrane antigens (EMA) was observed, while ER-α and PR A-B receptor isoforms were weakly expressed. Strong immunostaining with anti-CD10 and anti-vimentin antibodies was observed of the stromal component surrounding the ectopic glandular structures in both uteri. Likewise, intense expression of α-SMA and desmin proteins was revealed in the smooth muscle fibres adjacent to the foci of the misplaced glands along the myometria (Table 2).

## 3. Discussion

In the present study, focally and diffusely misplaced endometrial-type glands and stroma settled deep in the myometrium were histologically observed in 12 informative uterine tissues from a retrospective cohort composed of 420 female foetuses (2.8% of the cases). These findings suggest the occurrence of histogenetic changes during foetal uterine tissue development in the evaluated cases, with the various clinical characteristics reported in Table 1. Overall, the anatomical location, the incidence, and the histological parameters of the misplaced tissue depicting glandular, stromal, and smooth muscle cell hyperplasia support the existence of a not-yet-categorised developmental adenomyotic condition in the analysed foetal uteri.

Histologically, heterogeneous islands of ectopic endometrial-type glands showing hyperplastic features, located in different anatomical sites and at different depths in the foetal uterine myometrium, were revealed in all of the informative samples (Figure 1C). Likewise, an abundant periglandular stromal component exhibiting cellular hyperplasia and an enhanced microvascular network was observed in four different informative cases (foetal uteri cases no. 3, 5, 6, and 11) (Figure 1C,D). Additionally, distinct areas of disarrayed and hyperplastic smooth muscle cells adjacent to the misplaced glands or at distance from them were revealed in four informative cases (foetal uteri cases no. 3, 5, 6, and 7). Also, dense areas of fibrous bundles surrounding the ectopic glands were revealed in two separate informative cases (foetal uteri cases no. 7 and 8) (Figure 1F).

In general, the described histological characteristics of the displaced endometrial-type glandular foci in the foetal uterine wall appear to be remarkably evocative of the morphological features observed in uterine lesions in women with adenomyosis. Indeed, the adenomyotic lesions in patients are characterised by the presence of irregular nodules composed of histologically altered ectopic endometrial glands surrounded by a thickened stromal component with increased vascularity, infiltrating the myometrium at a distance from the JZ [28,29,30]. In addition, smooth muscle cell hyperplasia/hypertrophy, causing focal or diffuse myometrial thickening and uterine enlargement, is frequently observed [28,29]. In this regard, the focal smooth muscle hyperplasia and hypertrophy observed in association with areas of adenomyosis in adult women is mechanistically explained as a response of the myometrium to the presence of ectopic endometrial cells [1,22]. This argument could be a plausible mechanistic explanation concerning the stromal and smooth muscle hyperplasia observed in our study as a consequence of the ectopic endometrial cells spreading inside the foetal myometrium [1].

In addition, the fibrotic areas observed in the myometrium of two informative cases (foetal uteri cases no. 7, 8) strongly remind the authors of the fibrogenetic process frequently observed in adult adenomyosis [31]. In this respect, extensive fibrotic changes rather than endometrioid stroma with or without surrounding smooth muscle hyperplasia were observed in approximately 10% of 68 patients from a cohort who underwent laparoscopic treatment for adenomyosis [32]. It was also reported that such fibrotic changes vary widely and are mostly dependent on the adenomyosis subtype, classified as diffuse, sclerotic, nodular, or cystic [29,32].

The immunophenotypic analysis of the informative foetal uteri indicates a predominantly low to moderate level of expression of both ER and PR steroid receptors in the misplaced foetal endometrial glands and stroma, which is presumably related to the undifferentiated foetal tissues in general [33]. In this regard, it has previously been shown that the expression of estrogen receptor (ER-α) in the Müllerian duct and its derivatives, including the uterine fundus and the uterine corpus, varies on a cranial–caudal basis [34]. Indeed, these authors showed that the epithelium of the cranial part of the developing Müllerian duct expressed ER-α as early as at the eighth week of gestation, while the epithelium of the uterine corpus and uterine cervix examined at 12 to 21 weeks was negative for ER-α [34]. Comparatively, a high level of steroid receptor expression is observed in uterine tissues from adult patients affected by adenomyosis, confirming oestrogen-dependent pathogenesis [5,35].

An additional, significant finding is the presence of histologically variable ectopic glandular foci along the myometrium in the two individual monozygotic twins’ foetal uteri. The presence of ectopic endometrial tissue with remarkably similar morphology in both twins’ uteri firmly suggests a genetically driven process involved in foetal uterine tissue remodelling. In this context, a recent study using Mendelian randomisation analysis based on single-nucleotide polymorphisms identified several novel coding genes causally involved in the development of adult adenomyosis [36]. These data are in agreement with the former assumptions that the endometriotic disorder concomitantly occurring with adenomyosis in women is developed on a genetic basis [37,38]. In the same context, it was reported that genetic and epigenetic predispositions contribute to the pathogenesis of adenomyosis and endometriosis in adolescents [8,39]. In particular, candidate genes regulating oestrogen metabolism, inflammatory pathways, and Müllerian development, as well as epigenetic alterations, were delineated to be involved in the initiation of both adenomyosis and endometriosis in genetically susceptible patients [36,39,40].

Together, the presented findings suggest that the adenomyotic condition might be present from the foetal stage, reinforcing the embryogenetic theory and categorising the developmental form as a defect attributed to anomalies occurring during Mullerian duct development [7,9,10,12]. Likewise, the described observational approach can significantly contribute to better defining the putative genetic, epigenomic, environmental, or endocrine modifications involved in the pathogenesis of developmental adenomyosis [3,11].

The presented investigation provides a comprehensive analysis of misplaced endometrial-type glands and stroma in the myometrium of female foetal uteri examined after medical termination of pregnancy. The primary strength lies in the integrative analysis portraying the histogenetic changes in the uterine tissues from a large cohort evocative of a poorly categorised type of developmental adenomyosis. Moreover, the described analytical approach convenient for phenotypic characterisation of ectopic foetal uterine tissues may represent an experimental strategy for discovering novel tissue marker molecules for adenomyosis. In this respect, applying the contemporary multiplexed imaging technologies that enable detection and quantification of several molecular markers expressed in the misplaced endometrial foetal tissue might, importantly, highlight the pathogenesis of the adenomyotic disorder in general [41,42].

There are some limitations of the study, the principal difficulty being the possibility of defining the stage of morphogenesis at which to assess the presence of ectopic endometrial tissue in the myometrium. Another important element is the lack of follow-up in order to assess whether the presence of developmental adenomyotic changes in foetal uteri can cause disease in the postnatal period. At present, it is not possible to correlate these data with the subsequent development of the disease. The last limitation of the study is the estimation of the real incidence of the displaced endometrial tissue in the foetal uteri, considering the technical difficulties in preserving authentic tissue morphology during histopathological analytical procedures. Therefore, the reported incidence of ectopic glands in 2.8% of the informative cases analysed might be underestimated.

## 4. Materials and Methods

### 4.1. Fetal Uteri

The presence of displaced endometrial-type glands and stroma in the myometrium was evaluated in a retrospective cohort of 420 foetal uteri, including one twin pair, collected and categorised in the facility of the institutional pathology laboratory. The 12/420 foetal uteri cases selected showing ectopic endometrial tissue in this study are characterised genetically as shown in Table 1. The other 408/420 foetal uteri show similar malformations. The tissue samples were obtained after medically indicated termination of pregnancy (TOP), which was approved by the institutional Ethics Committee. On a regular basis at admission, written informed consent was obtained from each woman undergoing the pregnancy interruption procedure. The foetal uteri tissue samples were collected during an extended retroactive period with the aim to study the pathogenesis of adenomyotic disease. The study on the foetal tissues was conducted in accordance with the Declaration of Helsinki, and the protocol was approved by the local Ethics Committee.

### 4.2. Histopathological Analysis

At autopsy, foetal uteri were dissected from the pelvic organs, weighed, fixed in 10% buffered formalin, and embedded in paraffin. A particular tissue sampling procedure for the uteri was employed, i.e., between 100 and 200 sections with a thickness of 5–7 µm from each paraffin block containing uteri were prepared. To obtain tissue sections containing ectopic endometrial glands, every sixth slide was separately stained (H&E) and microscopically analysed. The foetal uteri that showed the presence of ectopic glands and stroma histologically evocative of adenomyotic disorder were considered as informative cases. Additionally, sections from the two individual foetal uteri from the monozygotic twin pair were prepared as 5–7 µm thick slices at step serial cycles at 200 µm intervals and evaluated for the presence of ectopic endometrial tissue.

### 4.3. Immunohistochemistry (IHC)

The paraffin-embedded foetal uteri from the informative cases were sectioned at 4–5 µm, deparaffinised in xylene, rehydrated through a graded alcohol series, and washed in PBS. The sections were then subjected to heat-induced epitope retrieval in citrate buffer (pH = 6) for 25 min at 96 °C and treated with proteinase-K, essentially as described in [27]. After a brief wash in PBS, the appropriate dilution of the primary antibodies was applied on slides for 1 h. Immunoreactivity was revealed using the avidin–biotin complex method (LSAB2 System HRP, Dako, Denmark) with 3,3′-diaminobenzidine tetrahydrochloride (DAB) as a chromogen. After checking the staining intensity, the sections were washed in water and counterstained with Harris haematoxylin (Sigma-Aldrich Chimie Sarl, Saint-Quentin Fallavier, France). Negative controls consisted of the replacement of the primary antibodies with non-immune mouse or rabbit serum or buffer alone. The extent and the intensity of the staining were determined by the objective observer procedure.

The staining intensity was graded on a scale of 0 to 3, where 0 meant no staining was assessed with anti-rabbit secondary antibody alone (negative); weak/neg equated to 0–5% positive cells; moderate/strong equated to 45–50% positive cells; and 3 meant there was strong staining, with more than 75% of cells stained. Tissue sections on slides were imaged using a Leica trinocular microscope DM4B and a Leica LAS X Navigator Software, Suite X application version (Leica Microsystems, Nanterre, France).

The following specific antibodies were employed: rabbit anti-oestrogen receptor-ER-α (ER-α) (Santa Cruz, CA, USA, cat no. sc-543), monoclonal mouse anti-human progesterone receptor, clone PgR 636 (Agilent/Dako, Les Ulis, France) detecting A and B forms; monoclonal mouse(Agilent/Dako, Les Ulis, anti-human pan-cytokeratin, clone MNF 116, reacting with cytokeratins 5, 6, 8, 17, and 19; anti-human EMA (epithelial membrane antigen), clone E29 (Agilent/Dako, Les Ulis, France); monoclonal mouse anti-human CD10, clone 56C6 (Agilent/Dako, Les Ulis,, France) and monoclonal mouse anti-vimentin, clone VIM 3B (Agiulent/Dako, Les Ulis, France): anti-α-Actin (α-Smooth Muscle Actin) antibody, clone 1A4 (Agilent/Dako, Les Ulis France); and mouse anti-human Desmin, clone 033 (CliniSciences, Les Ulis, France). The immunoreactivity was revealed with either rabbit anti-mouse or goat anti-rabbit IgG peroxidase-conjugated antibodies (LSAB2 system, Dako, Glostrup, Denmark) according to the provider’s instructions.

## 5. Conclusions

The presented results confirmed the presence of developmentally misplaced endometrial tissue in the myometrium of twelve foetal uteri from a large cohort composed of 420 cases. The observed glandular, stromal, and smooth muscle cell hyperplastic changes consistently delineate a specific histogenetic mechanism involved in foetal uterine development. In parallel, the histomorphological finding of misplaced glandular structures disposed as cords lengthwise in the myometrium in the two monozygotic twins’ uteri further emphasises the genetically controlled tissue organisation process. Concerning the clinical importance, the developmental perspective could explain the occurrence of adenomyosis in women without prior uterine injury or surgery, highlighting the role of congenital factors in this disease pathogenesis. Finally, recognising adenomyosis likewise as a developmental disease can open novel preventive and therapeutic strategies aimed at mitigating disease impact before clinical manifestation.

## Figures and Tables

**Table 1:** Summary of the clinical characteristics of the twelve (12) informative foetuses exhibiting developmentally displaced glands and stroma in the myometrium.

Foetus Case No.	WP	IUFD/TOP	Karyotype	Clinical Findings	G/P
1	18	TOP	Normal	Osteogenesis imperfecta	G2/P0
2	19	TOP	Trisomy 18	Growth retardation, Facial dysmorphia, Camptodactyly	G1
3	24	TOP	Normal	Cerebral anomalies	G1
4	24	TOP	Normal	Foetal akinesia deformation sequence	G1
5	25	TOP	Normal	Bilateral renal agenesis	G1
6	25.2	IUFD	N.D.	Placental vascular pathology	G4/P3
7	26.5	TOP	Normal	Heart malformation, Clefting	G1
8	27	IUFD	Normal	PRS, Adactyly/Syndactyly	G1
9	28	IUFD	N.D.	TTTS	G2/P1
10	28	IUFD	N.D.		
11	34	TOP	Normal	Cerebral anomalies	G5/P0
12	37.4	Neonatal death	Normal	Congenital diaphragmatic hernia	N.d.

N.D.: not done; N.d.: not documented; Foetus Case No.: Foetus case number; WP: weeks of pregnancy/gestation age; TOP: termination of pregnancy; IUFD: in utero foetal death; G/P: gravidity/parity; PRS: Pierre Robin syndrome; TTTS: twin-to-twin transfusion syndrome.

**Table 2:** Evaluation of IHC staining of the twelve informative uteri with the selected series of specific antibodies.

Informative Uterus No.	EMA		Cytokeratin		ER		PR		Vimentin		CD10	
	GL	St	GL	St	GL	St	GL	St	GL	St	GL	St
1	strong	neg	strong	neg	weak	weak	moderate	moderate/strong	neg	strong	neg	strong
2	strong	neg	strong	neg	weak	weak	moderate	moderate/strong	neg	strong	neg	strong
3	strong	neg	strong	neg	weak	weak	moderate	moderate/strong	neg	strong	neg	strong
4	strong	neg	strong	neg	weak	weak	moderate	moderate/strong	neg	strong	neg	strong
5	strong	neg	strong	neg	weak	weak	moderate	moderate/strong	neg	strong	neg	strong
6	strong	neg	strong	neg	N.D.	N.D.	moderate	moderate/strong	neg	strong	neg	strong
7	strong	neg	strong	neg	weak	weak	moderate	moderate/strong	neg	strong	neg	strong
8	strong	neg	strong	neg	weak	weak	moderate	moderate/strong	neg	strong	neg	strong
9	strong	neg	strong	neg	weak	weak	neg	neg	neg	strong	neg	strong
10	strong	neg	strong	neg	weak	weak	neg	neg	neg	strong	neg	strong
11	strong	neg	strong	neg	weak	weak	moderate	moderate/strong	neg	strong	neg	strong
12	strong	neg	strong	neg	weak	weak	moderate	moderate/strong	N.D.	N.D.	neg	strong

N.D.: not done; EMA: epithelial membrane antigen; ER: oestrogen receptor; PR: progesterone receptor A and B isoforms; St: stroma; GL: glands; neg: negative. Weak/neg: 0–5% of stained cells; moderate/strong: 45–50% of stained cells; strong: more than 70% of stained cells.

## Data Availability

The original contributions presented in this study are included in the article. Further inquiries can be directed to the corresponding authors.

## Abbreviations

The following abbreviations are used in this manuscript:

ER	oestrogen receptor
PR	progesterone receptor
mm	millimetres
N.D.	not done
N.d.	not documented
WP	weeks of pregnancy
TOP	termination of pregnancy
IUFD	in utero foetal death
JZ	junctional zone
G/P	gravidity/parity
PRS	Pierre Robin syndrome
TTTS	twin-to-twin transfusion syndrome
H&E	haematoxylin and eosin
EMA	epithelial membrane antigen
St	stroma
GL	glands
neg	negative
α-SMA	alpha-Smooth Muscle Actin

## References

[1] Ferenczy A. (1998). Pathophysiology of adenomyosis. Hum. Reprod. Update.

[2] Leyendecker G., Wildt L., Mall G. (2009). The pathophysiology of endometriosis and adenomyosis: Tissue injury and repair. Arch. Gynecol. Obstet..

[3] Zhai J., Vannuccini S., Petraglia F., Giudice L.C. (2020). Adenomyosis; Mechanisms and pathogenesis. Semin. Reprod. Med..

[4] Stratopoulou C.A., Donnez J., Dolmans M.M. (2021). Origin and Pathogenic Mechanisms of Uterine Adenomyosis: What is known so far. Reprod. Sci..

[5] García-Solares J., Donnez J., Donnez O., Dolmans M.M. (2018). Pathogenesis of uterine adenomyosis: Invagination or metaplasia? Fertil. Steril..

[6] Emge L.A. (1962). The elusive adenomyosis of the uterus. Its historical past and its present state of recognition. Am. J. Obstet. Gynecol..

[7] Batt R.E., Yeh J. (2013). Müllerianosis: Four developmental (embryonic) mullerian diseases. Reprod. Sci..

[8] Bulun S.E., Yildiz S., Adli M., Chakravarti D., Parker J.B., Milad M., Yang L., Chaudhari A., Tsai S., Wei J.J. (2023). Endometriosis and adenomyosis: Shared pathophysiology. Fertil. Steril..

[9] Signorile P.G., Baldi A. (2010). Endometriosis: New concepts in the pathogenesis. Int. J. Biochem. Cell Biol..

[10] Signorile P.G., Baldi A., Viceconte R., Boccellino M. (2025). The Role of Adenogenesis Factors in the Pathogenesis of Endometriosis. Int. J. Mol. Sci..

[11] Giudice L.C., Liu B., Irwin J.C. (2025). Endometriosis and adenomyosis unveiled through single-cell glasses. Am. J. Obstet. Gynecol..

[12] Bouquet de Jolinière J., Ayoubi J.M., Lesec G., Validire P., Goguin A., Gianaroli L., Dubuisson J.B., Feki A., Gogusev J. (2012). Identification of Displaced Endometrial Glands and Embryonic Duct Remnants in Female Fetal Reproductive Tract: Possible Pathogenetic Role in Endometriotic and Pelvic Neoplastic Processes. Front. Physiol..

[13] Huseby R.A., Thurlow S. (1982). Effects of prenatal exposure of mice to “low dose” dyethylstilbestroland the development of adenomyosis associated with evidence of hyperprolactinemia. Am. J. Obstet. Gynecol..

[14] Stephens V.R., Rumph J.T., Ameli S., Bruner-Tran K.L., Osteen K.G. (2022). The Potential Relationship Between Environmental Endocrine Disruptor Exposure and the Development of Endometriosis and Adenomyosis. Front. Physiol..

[15] Newbold R.R., Jefferson W.N., Padilla-Banks E. (2007). Long-term adverse effects of neonatal exposure to bisphenol A on the murine female reproductive tract. Reprod. Toxicol..

[16] Benagiano G., Habiba M., Brosens I. (2012). The pathophysiology of uterine adenomyosis: An update. Fertil. Steril..

[17] Habiba M., Benagiano G. (2021). Classifying Adenomyosis: Progress and Challenges. Int. J. Environ. Res. Public Health.

[18] Habiba M., Guo S.W., Benagiano G. (2023). Are Adenomyosis and Endometriosis Phenotypes of the Same Disease Process?. Biomolecules.

[19] Munro M.G. (2020). Classification and Reporting Systems for Adenomyosis. J. Minim. Invasive Gynecol..

[20] Donnez J., Donnez O., Dolmans M.M. (2018). Introduction: Uterine adenomyosis, another enigmatic disease of our time. Fertil. Steril..

[21] Moawad G., Fruscalzo A., Youssef Y., Kheil M., Tawil T., Nehme J., Pirtea P., Guani B., Afaneh H., Ayoubi J.M. (2023). Adenomyosis: An Updated Review on Diagnosis and Classification. J. Clin. Med..

[22] Reinhold C., Tafazoli F., Mehio A., Wang L., Atri M., Siegelman E.S., Rohoman L. (1999). Uterine adenomyosis: Endovaginal US and MR imaging features with histopathologic correlation. Radiographics.

[23] Kishi Y., Suginami H., Kuramori R., Yabuta M., Suginami R., Taniguchi F. (2012). Four subtypes of adenomyosis assessed by magnetic resonance imaging and their specification. Am. J. Obstet. Gynecol..

[24] Bird C.C., McElin T.W., Manalo-Estrella P. (1972). The elusive adenomyosis of the uterus—Revisited. Am. J. Obstet. Gynecol..

[25] Azziz R. (1989). Adenomyosis: Current perspectives. Obstet. Gynecol. Clin. N. Am..

[26] Lee N.C., Dicker R.C., Rubin G.L., Ory H.W. (1984). Confirmation of the preoperative diagnoses for hysterectomy. Am. J. Obstet. Gynecol..

[27] Hsu S.M., Raine L., Fanger H. (1981). The use of antiavidin antibody and avidin-biotin-peroxidase complex in immunoperoxidase technics. Am. J. Clin. Pathol..

[28] Antero M.F., Ayhan A., Segars J., Shih I.M. (2020). Pathology and Pathogenesis of Adenomyosis. Semin. Reprod. Med..

[29] Liu Z., Guo Y., Pan X., Liu G., Yang X. (2023). Histopathological characteristics of adenomyosis: Structure and microstructure. Histol. Histopathol..

[30] Levgur M., Abadi M.A., Tucker A. (2000). Adenomyosis: Symptoms, Histology, and Pregnancy Terminations. Obstet. Gynecol..

[31] Huang Q., Liu X., Critchley H., Fu Z., Guo S.W. (2022). How does the extent of fibrosis in adenomyosis lesions contribute to heavy menstrual bleeding?. Reprod. Med. Biol..

[32] Pistofidis G., Makrakis E., Koukoura O., Bardis N., Balinakos P., Anaf V. (2014). Distinct types of uterine adenomyosis based on laparoscopic and histopathologic criteria. Clin. Exp. Obstet. Gynecol..

[33] Takeyama J., Suzuki T., Inoue S., Kaneko C., Nagura H., Harada N., Sasano H. (2001). Expression and cellular localization of estrogen receptors α and β in the human fetus. J. Clin. Endocrinol. Metab..

[34] Cunha G., Li Y., Mei C., Derpinghaus A., Baskin L. (2021). Ontogeny of estrogen receptors in human male and female fetal reproductive tracts. Differentiation.

[35] Kitawaki J. (2006). Adenomyosis: The pathophysiology of an oestrogen-dependent disease. Best Pract. Res. Clin. Obstet. Gynaecol..

[36] Yang Q., Zhong F., Liu X., Hong J., Chen L., Lin H., Fu J., Zheng X., Jiang J. (2025). Identification of novel causally related genes in adenomyosis: An integrated summary data-based Mendelian randomization study and bioinformatics analysis. Medicine.

[37] Moen M.H. (1994). Endometriosis in monozygotic twins. Acta Obstet. Gynecol. Scand..

[38] Hadfield R.M., Mardon H.J., Barlow D.H., Kennedy S.H. (1997). Endometriosis in monozygotic twins. Fertil. Steril..

[39] Palumbo M., Della Corte L., Ascione M., D’Angelo G., Colacurci D., Baldini G.M., Pellicano M., Giampaolino P., Bifulco G. (2025). Genetic and Epigenetic Components in the Pathogenesis of Adenomyosis and Endometriosis in Adolescents. Biomedicines.

[40] Giacomini E., Minetto S., Li Piani L., Pagliardini L., Somigliana E., Vigano P. (2021). Genetics and Inflammation in Endometriosis: Improving Knowledge for Development of New Pharmacological Strategies. Int. J. Mol. Sci..

[41] Black S., Phillips D., Hickey J.W., Kennedy-Darling J., Venkataraaman V.G., Samusik N., Goltsev Y., Schürch C.M., Nolan G.P. (2021). CODEX multiplexed tissue imaging with DNA-conjugated antibodies. Nat. Protoc..

[42] Harms P.W., Frankel T.L., Moutafi M., Rao A., Rimm D.L., Taube J.M., Thomas D., Chan M.P., Pantanowitz L. (2023). Multiplex Immunohistochemistry and Immunofluorescence: A Practical Update for Pathologists. Mod. Pathol..

